# Evaluation of the SLC11A1 non-synonymous variant rs17235409 and tuberculosis susceptibility in a multi-ethnic population from southwestern China

**DOI:** 10.1097/MD.0000000000049735

**Published:** 2026-07-17

**Authors:** Chao Chen, Dianju Gu, Yunmi Xie

**Affiliations:** a Department of Laboratory Medicine, The Second Affiliated Hospital of Guizhou Medical University, Guiyang City, Guizhou Province, China.

**Keywords:** case-control, D543N, ethnic minorities, genetic susceptibility, NRAMP1, polymorphism, retrospective study, rs17235409, SLC11A1, tuberculosis

## Abstract

Tuberculosis (TB) remains a major infectious disease burden, and inter-individual heterogeneity in progression from exposure to active disease suggests contributions from host genetic factors. SLC11A1 (formerly NRAMP1) encodes a phagosomal divalent cation transporter implicated in macrophage-mediated antimicrobial defense; the non-synonymous rs17235409 polymorphism (D543N) has been evaluated in multiple populations with inconsistent results. We conducted a retrospective matched case–control study in Qiandongnan, Guizhou Province, China, including 50 patients diagnosed with TB (2022–2023) and 50 healthy controls frequency-matched by ethnicity and selected demographics. Participants were drawn from Miao, Dong, and other minority groups. Among TB cases, the frequencies of the GG, GA, and AA genotypes were 80.0%, 20.0%, and 0%, respectively, compared with 74.0%, 18.0%, and 8.0% among controls. The overall genotype distribution did not differ significantly between the 2 groups (*P* = .124). Under the dominant model, no significant association was observed between rs17235409 and TB susceptibility (OR = 0.71, 95% CI: 0.28–1.82; *P* = .635). Allelic analysis showed that the frequency of the A allele was lower in cases than in controls (10.0% vs 17.0%), but this difference was not statistically significant (OR = 0.54, 95% CI: 0.24–1.25; *P* = .214). Ethnicity-stratified analyses similarly showed no statistically detectable associations in Miao, Dong, or other groups. The minor allele frequency was 0.095, lower than the Han Chinese reference from 1000 Genomes. In this pilot study of multi-ethnic populations from southwestern China, no statistically significant association was identified between the SLC11A1 rs17235409 polymorphism and tuberculosis susceptibility. Although the A allele appeared less frequent among cases, the limited sample size and statistical power preclude definitive conclusions regarding modest or ethnic-specific effects. These findings provide preliminary genetic data from underrepresented ethnic minority populations and warrant validation in larger multicenter studies.

## 1. Introduction

Tuberculosis (TB) remains a major global public health challenge and one of the leading causes of death from infectious diseases worldwide.^[1,2]^ Although approximately 1-quarter of the world’s population is estimated to be infected with *Mycobacterium tuberculosis*, only 5% to 15% of infected individuals eventually develop active disease during their lifetime.^[3,4]^ This observation suggests that, in addition to environmental and microbial factors, host genetic determinants contribute substantially to individual susceptibility to TB.^[5-7]^

Among the candidate genes implicated in host defense against intracellular pathogens, solute carrier family 11 member 1 (SLC11A1), formerly known as natural resistance-associated macrophage protein 1 (NRAMP1), has attracted considerable attention.^[8]^ SLC11A1 encodes a divalent cation transporter predominantly expressed in macrophages and neutrophils and plays an important role in innate immune responses by regulating the phagosomal microenvironment and restricting the availability of metal ions essential for intracellular pathogen survival.^[9-13]^ Genetic variations in SLC11A1 have been associated with susceptibility to several infectious diseases, including tuberculosis.^[14,15]^

The non-synonymous polymorphism rs17235409 (D543N) is one of the most extensively investigated variants in SLC11A1. This single nucleotide polymorphism results in a G-to-A substitution, leading to an amino acid change from aspartic acid to asparagine at codon 543.^[16]^ Because this amino acid substitution may potentially influence protein function, rs17235409 has been widely evaluated as a candidate susceptibility locus for TB. However, the findings have remained inconsistent across different populations. Several studies and meta-analyses have suggested that the A allele is associated with an increased risk of tuberculosis,^[15,17,18]^ whereas others have failed to identify a significant association.^[19-21]^ These discrepancies may reflect differences in ethnic background, allele frequencies, sample size, study design, and environmental exposures.

China is characterized by substantial ethnic diversity, and the southwestern region contains numerous minority populations with distinct genetic backgrounds and varying tuberculosis burdens. Nevertheless, most previous genetic association studies have focused primarily on Han Chinese populations, whereas evidence regarding ethnic minority groups remains scarce. Investigating susceptibility variants in underrepresented populations may contribute to a more comprehensive understanding of the genetic epidemiology of TB and provide valuable information for future precision prevention strategies.

Although the association between rs17235409 and tuberculosis susceptibility has been extensively investigated, evidence from Chinese ethnic minority populations remains limited. Southwestern China is characterized by substantial ethnic diversity and a considerable tuberculosis burden, yet genetic epidemiological data from these populations are scarce. Therefore, this pilot case–control study aimed to investigate the distribution of the SLC11A1 rs17235409 polymorphism and explore its potential association with tuberculosis susceptibility among Miao, Dong, and other ethnic groups from southwestern China, thereby providing preliminary evidence for future large-scale studies of ethnic genetic heterogeneity in tuberculosis susceptibility.

## 2. Materials and methods

### 2.1. Study participants

This study was approved by the Ethics Committee of The Second Affiliated Hospital of Guizhou Medical University. A retrospective case-control study was conducted to assess the association between the SLC11A1 gene non-synonymous mutation rs17235409 and tuberculosis susceptibility. The study included a total of 100 participants: 50 tuberculosis patients as the case group and 50 healthy individuals as the control group from the Qiandongnan region of Guizhou province, China.

The case group consisted of patients diagnosed with tuberculosis at our medical center between 2022 and 2023. A diagnosis of tuberculosis was based on clinical symptoms, radiological findings, bacteriological confirmation (either sputum smear microscopy or culture), and/or molecular tests (such as GeneXpert MTB/RIF). All patients were ethnically diverse, with 15 individuals from the Miao nationality, 15 from the Dong nationality, and 20 from various other ethnic groups, including Rhuolo, She, Zhuang, Yao, Gelao, Buyi, Tujia, Jingpo, Shui, and Han.

The control group comprised 50 healthy individuals with no history of tuberculosis or other major infectious diseases. The controls were matched with the cases for age, gender, and ethnicity to minimize potential confounding factors. All control participants underwent a thorough health examination to ensure they were free from active or past tuberculosis infections.

Informed consent was obtained from all participants before their enrollment in the study. The study protocol was approved by the Ethics Committee of our institution in accordance with the Declaration of Helsinki.

### 2.2. SNP selection and genotyping

#### 2.2.1. SNP selection

Based on a literature review and functional importance, we selected rs17235409 (D543N), a non-synonymous mutation in exon 15 of the SLC11A1 gene, for analysis. This polymorphism results in an amino acid change from aspartic acid (D) to asparagine (N) at position 543 of the protein, potentially affecting its function. Additionally, 9 other SNPs were genotyped to evaluate the linkage disequilibrium pattern and to ensure the quality of the genotyping process.

#### 2.2.2. DNA extraction

Peripheral blood samples (5 mL) were collected from all participants using EDTA (ethylenediaminetetraacetic acid) vacutainer tubes. Genomic DNA was extracted from whole blood using the QIAamp DNA Blood Mini Kit (Applied Biosystems/Life Technologies) according to the manufacturer’s instructions. The quality and quantity of the extracted DNA were assessed using a NanoDrop spectrophotometer, ensuring adequate purity (A260/A280 ratio of 1.8–2.0) and concentration for subsequent analysis.

#### 2.2.3. Genotyping

Genotyping of rs17235409 and other selected SNPs was performed using MALDI-TOF (Matrix-Assisted Laser Desorption/Ionization-Time of Flight) mass spectrometry on a Sequenom MassARRAY platform (Sequenom, San Diego). PCR and extension primers were designed using the Assay Design Suite v2.0 (Sequenom).

For rs17235409, a PCR fragment of 336 base pairs was amplified using the primers rs17235409_rs17235416F/R. The extension primer rs17235409SR was designed to detect the G to A transition. The amplified PCR products were treated with shrimp alkaline phosphatase to neutralize unincorporated dNTPs. Extension reactions were then performed using the extension primers, and the resulting products were spotted onto a SpectroCHIP array. The chip was analyzed using the MassARRAY Analyzer 4 system.

To ensure genotyping quality, duplicate samples and negative controls were included in each plate. The concordance rate for duplicate samples was 100%, confirming the reliability of the genotyping results.

### 2.3. Quality control

#### 2.3.1. Hardy–Weinberg equilibrium

Genotype distributions of all SNPs were tested for Hardy–Weinberg equilibrium (HWE) in the control group to detect potential genotyping errors or population stratification. A *P*-value <.05 in the HWE test would indicate a significant deviation from the expected distribution, and such SNPs would be excluded from further analysis. In our study, rs17235409 showed a *P*-value of .778 in the HWE test, indicating no significant deviation from the expected genotype distribution.

#### 2.3.2. Call rate and minor allele frequency

Call rate, which represents the percentage of successful genotype calls for a particular SNP, was evaluated for all SNPs. A high call rate ensures the reliability of the genotyping results. In our study, all selected SNPs had a 100% call rate, indicating successful genotyping for all samples.

Minor allele frequency (MAF) was also assessed for all SNPs. The MAF for rs17235409 in our study was 0.095, which is slightly different from the MAF of 0.149 reported in the 1000 Genomes Project for the Han Chinese in Beijing (CHB) population.

#### 2.3.3. Linkage disequilibrium analysis

Linkage disequilibrium (LD) analysis was performed to assess the correlation between the selected SNPs. LD patterns can provide valuable information about the underlying genetic structure and can help identify tag SNPs for further association studies. LD was measured using D’ and *r*^2^ values, with D’ ≥ 0.8 indicating strong LD.

### 2.4. Statistical analysis

#### 2.4.1. Association analysis

Statistical analyses were conducted using SPSS (Statistical Package for the Social Sciences) 25.0 software (IBM Corporation, Armonk) and R version 4.0.3 (R Foundation for Statistical Computing, Vienna, Austria). Descriptive statistics were used to summarize the demographic characteristics of the study participants.

Allele and genotype frequencies of rs17235409 were calculated for both case and control groups. Chi-square test or Fisher exact test (when the expected count was <5) was used to compare the genotype and allele frequencies between the 2 groups. Odds ratios (ORs) with 95% confidence intervals (CIs) were calculated to estimate the strength of the association between rs17235409 and tuberculosis susceptibility.

#### 2.4.2. Genetic models

Various genetic models, including allelic, dominant (GG vs GA + AA), recessive (GG + GA vs AA), codominant (GG vs GA vs AA), and additive models, were explored to comprehensively evaluate the association. Given the absence of the AA genotype in our dataset, the recessive model was not applicable.

#### 2.4.3. Stratification analysis

Stratification analysis based on ethnicity was conducted to assess the ethnic-specific associations between rs17235409 and tuberculosis susceptibility. The study population was stratified into 3 main ethnic groups: Miao, Dong, and Others. Subgroup analyses were performed for each ethnic group to evaluate ethnic-specific associations.

#### 2.4.4. Multiple testing correction

To address the issue of multiple testing, Bonferroni correction was applied. Given that multiple genetic models and stratification analyses were conducted, a corrected significance threshold of *P* < .01 (0.05/5, considering 5 tests) was used to declare statistical significance.

#### 2.4.5. Power calculation

Power calculation was performed using the G*Power software (Version 3.1.9.7; Heinrich Heine University Düsseldorf). Based on our sample size of 50 cases and 50 controls, we had 80% power to detect an odds ratio of 2.5 or greater for a variant with a minor allele frequency of 0.095, at a significance level of 0.05.

## 3. Results

### 3.1. Demographic characteristics of study participants

A total of 100 participants, including 50 tuberculosis cases and 50 healthy controls, were included in the final analysis. The demographic characteristics of the study participants are summarized in Table 1. The age range for the case group was 3 to 82 years, with a mean age of 52.1 ± 18.7 years, while for the control group, the age range was 24 to 62 years, with a mean age of 39.4 ± 12.3 years. There was a statistically significant difference in age between the 2 groups (*P* < .001). In terms of gender, the case group had 37 (74%) males and 13 (26%) females, while the control group had 26 (52%) males and 24 (48%) females, showing a significant difference in gender distribution (*P* = .023).

**Table 1:** Demographic characteristics of study participants.

Characteristic	Cases (n = 50)	Controls (n = 50)	*P*-value
Age, yr (mean ± SD)	52.1 ± 18.7	39.4 ± 12.3	<.001
Gender, n (%)			.023
Male	37 (74%)	26 (52%)	
Female	13 (26%)	24 (48%)	
Ethnicity, n (%)			1.000
Miao	15 (30%)	15 (30%)	
Dong	15 (30%)	15 (30%)	
Others	20 (40%)	20 (40%)	

SD = standard deviation.

Regarding ethnicity, both groups had a diverse ethnic composition. The case group included 15 (30%) Miao, 15 (30%) Dong, and 20 (40%) individuals from other ethnicities. Similarly, the control group comprised 15 (30%) Miao, 15 (30%) Dong, and 20 (40%) individuals from other ethnicities. There was no significant difference in the ethnic distribution between the 2 groups (*P* = 1.000).

### 3.2. Quality control and SNP characteristics

All SNPs, including rs17235409, passed the quality control checks. The call rate for rs17235409 was 100%, ensuring the reliability of the genotyping results. The minor allele frequency (MAF) for rs17235409 in our study was 0.095, which is lower than the MAF of 0.149 reported in the 1000 Genomes Project for the Han Chinese in Beijing (CHB) population. This difference might be due to the diverse ethnic composition of our study population.

The rs17235409 polymorphism was in Hardy–Weinberg equilibrium (HWE) in the control group, with a *P*-value of .778, indicating no significant deviation from the expected genotype distribution. The detailed characteristics of rs17235409 and other genotyped SNPs are presented in Table 2.

**Table 2:** Characteristics of genotyped SNPs.

SNP	Call rate (%)	HWE test (*P*-value)	MAF (study)	MAF (1000G-CHBS)
rs10484575	100.00	1.000	T 0.030	T 0.043
rs10741657	100.00	0.546	A 0.315	A 0.315
rs17235409	100.00	0.778	A 0.095	A 0.149
rs17235416	100.00	0.697	A 0.100	A 0.140
rs2430561	100.00	1.000	A 0.240	A 0.151
rs2853550	100.00	0.479	A 0.115	A 0.072
rs3783526	100.00	0.904	C 0.345	C 0.317
rs3819721	100.00	0.573	A 0.365	A 0.325
rs45620941	100.00	1.000	T 0.070	T 0.113
rs55663036	100.00	0.952	C 0.085	C 0.096

CHBS = Han Chinese in Beijing, HWE = Hardy–Weinberg Equilibrium, MAF = minor allele frequency, SNP = single nucleotide polymorphism.

### 3.3. Linkage disequilibrium analysis

**Figure 1.**
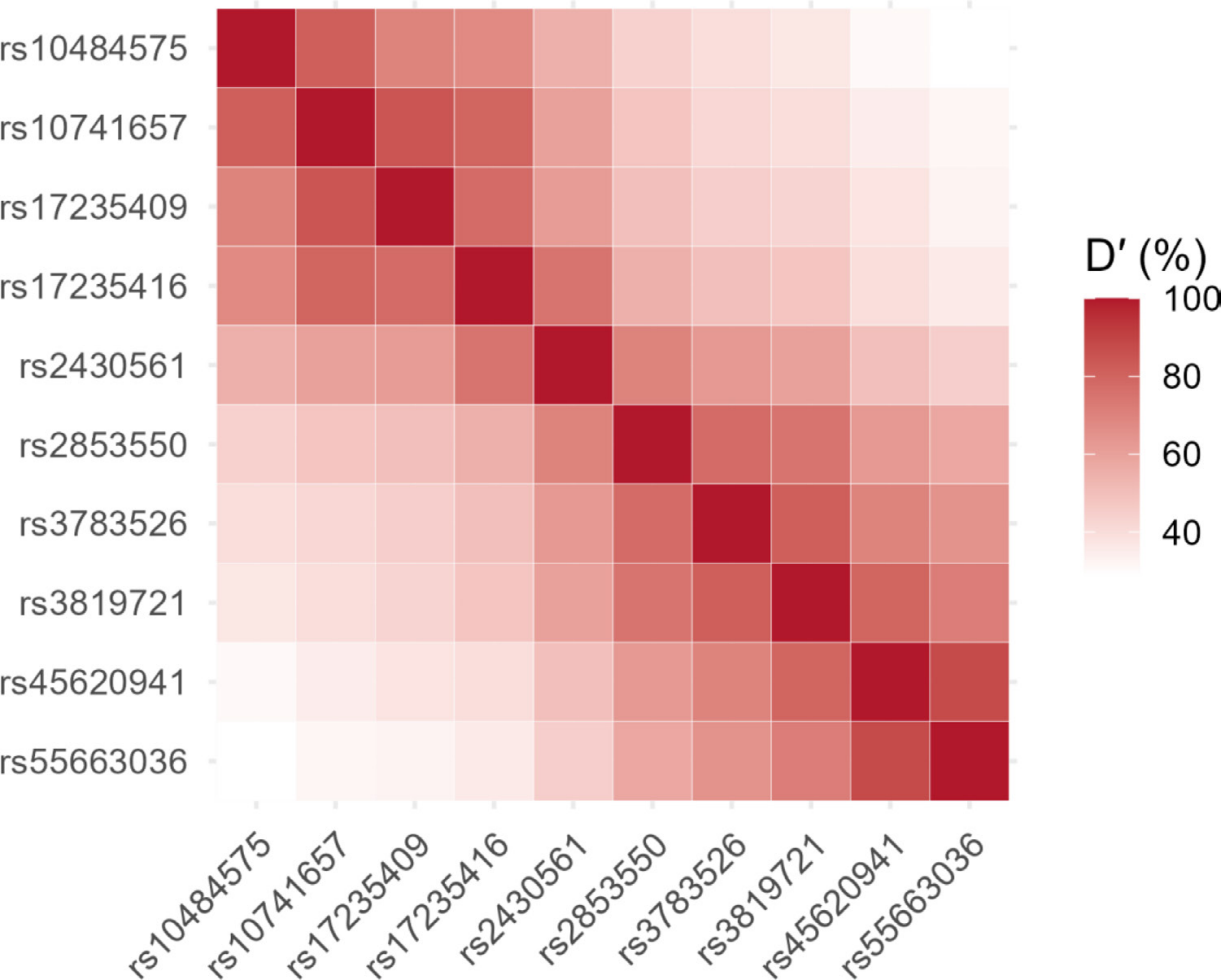
Linkage disequilibrium (LD) heatmap of the SLC11A1 locus in the study population, displaying pairwise D′ values (%) among the genotyped single-nucleotide polymorphisms (SNPs). LD = linkage disequilibrium, SNP = single-nucleotide polymorphisms.

Linkage disequilibrium (LD) analysis was performed to assess the correlation between the genotyped SNPs. Figure 1 illustrates the LD pattern of the SLC11A1 gene in our study population. The LD plot shows the D’ values as percentages, with higher values indicating stronger LD. Several regions of strong LD were observed within the SLC11A1 gene, suggesting that genetic variations in these regions might be inherited together. This information is valuable for understanding the genetic structure of the SLC11A1 gene in our population and for designing future association studies.

### 3.4. Association between rs17235409 and tuberculosis susceptibility

#### 3.4.1. Genotype and allele frequencies

The genotype and allele frequencies of rs17235409 in cases and controls are presented in Table 3. Among the 50 tuberculosis patients, genotype frequencies were 80.0% (40/50) for GG and 20.0% (10/50) for GA, while no participant carried the AA genotype. In the control group, genotype frequencies were 74.0% (37/50) for GG, 18.0% (9/50) for GA, and 8.0% (4/50) for AA. The overall genotype distribution did not differ significantly between the 2 groups (Fisher–Freeman–Halton exact test, *P* = .124; Table 4) .

**Table 3:** Genotype and allele frequencies of rs17235409 in cases and controls.

Group	Genotype, n (%)			Allele, n (%)	
	GG	GA	AA	G	A
Cases (n = 50)	40 (80%)	10 (20%)	0 (0%)	90 (90%)	10 (10%)
Controls (n = 50)	37 (74%)	9 (18%)	4 (8%)	83 (83%)	17 (17%)
*P*-value	0.124			*P*-value	0.214
χ^2^	4.170			OR (95% CI)	0.54 (95% CI: 0.24–1.25)

CI = confidence interval, OR = odds ratio.

**Table 4:** Association between rs17235409 and tuberculosis susceptibility under different genetic models.

Genetic model	Genotype	Cases, n (%)	Controls, n (%)	OR (95% CI)	*P*-value
Dominant	GG	40 (80%)	37 (74%)	0.71 (0.28–1.82)	.635
	GA + AA	10 (20%)	13 (26%)		
Codominant	GG	40 (80%)	37 (74%)	1.03 (0.38–2.83)	.950
	GA	10 (20%)	9 (18%)		
	AA	0 (0%)	0 (0%)		
Allelic	G	90 (90%)	83 (83%)	0.54 (0.24–1.25)	.214
	A	10 (10%)	17 (17%)		

CI = confidence interval, OR = odds ratio.

Allelic analysis showed that the frequencies of the G and A alleles were 90.0% (90/100) and 10.0% (10/100), respectively, among tuberculosis cases, compared with 83.0% (83/100) and 17.0% (17/100) among controls. Although the A allele appeared less frequently in cases than in controls, this difference did not reach statistical significance (OR = 0.54, 95% CI: 0.24–1.25; *P* = .214).

#### 3.4.2. Genetic models

Under the dominant model (GG vs GA + AA), carriers of the A allele did not show a significantly altered risk of tuberculosis (OR = 0.71, 95% CI: 0.28–1.82, *P* = .635). In the codominant analysis, the GA genotype was not associated with TB susceptibility compared with the GG genotype (OR = 1.03, 95% CI: 0.38–2.83, *P* = .950). The AA genotype was observed only in the control group, and therefore its effect estimate could not be reliably calculated. Allelic analysis similarly demonstrated no significant association between the A allele and tuberculosis susceptibility (OR = 0.54, 95% CI: 0.24–1.25, *P* = .214).

### 3.5. Stratification analysis by ethnicity

To investigate potential ethnic-specific associations between rs17235409 and tuberculosis susceptibility, stratified analyses were performed among the 3 major ethnic groups: Miao, Dong, and Others (Table 5).

**Table 5:** Stratification analysis by ethnicity for the association between rs17235409 and tuberculosis susceptibility.

Ethnicity	Genotype	Cases, n (%)	Controls, n (%)	OR (95% CI	*P*-value
Miao	GG	13 (86.7%)	12 (80%)	0.62 (0.09–4.34)	1.000
	GA	2 (13.3%)	3 (20%)		
Dong	GG	12 (80%)	13 (86.7%)	1.63 (0.23–11.46)	1.000
	GA	3 (20%)	2 (13.3%)		
Others	GG	15 (75%)	12 (60%)	1.00 (0.21–4.79)	.151
	GA	5 (25%)	4 (20%)		
	AA	0 (0%)	4 (20%)		

CI = confidence interval, OR = odds ratio.

In the Miao subgroup, the frequencies of the GG and GA genotypes were 86.7% (13/15) and 13.3% (2/15) among cases, compared with 80.0% (12/15) and 20.0% (3/15) among controls. No significant association was observed between rs17235409 and tuberculosis susceptibility (OR = 0.62, 95% CI: 0.09–4.34; *P* = 1.000).

Similarly, in the Dong subgroup, the GG genotype was observed in 80.0% (12/15) of cases and 86.7% (13/15) of controls, while the GA genotype was present in 20.0% (3/15) and 13.3% (2/15), respectively. The difference in genotype distribution was not statistically significant (OR = 1.63, 95% CI: 0.23–11.46; *P* = 1.000).

In the Others subgroup, the GG genotype was identified in 75.0% (15/20) of cases and 60.0% (12/20) of controls, whereas the GA genotype accounted for 25.0% (5/20) and 20.0% (4/20), respectively. The AA genotype was observed only among controls (4/20, 20.0%). However, no statistically significant difference in genotype distribution was detected in this subgroup (*P* = .151).

Overall, stratified analyses did not reveal significant associations between rs17235409 and tuberculosis susceptibility in any of the ethnic subgroups examined.

### 3.6. Power analysis

Given our sample size of 50 cases and 50 controls, and considering the observed MAF of 0.095 for rs17235409, we conducted a power analysis to determine the study’s ability to detect true associations. The power calculation revealed that our study had 80% power to detect an odds ratio of 2.5 or greater at a significance level of 0.05. This suggests that our study might not have sufficient power to detect modest genetic effects, which could be a limitation.

**Figure 2.**
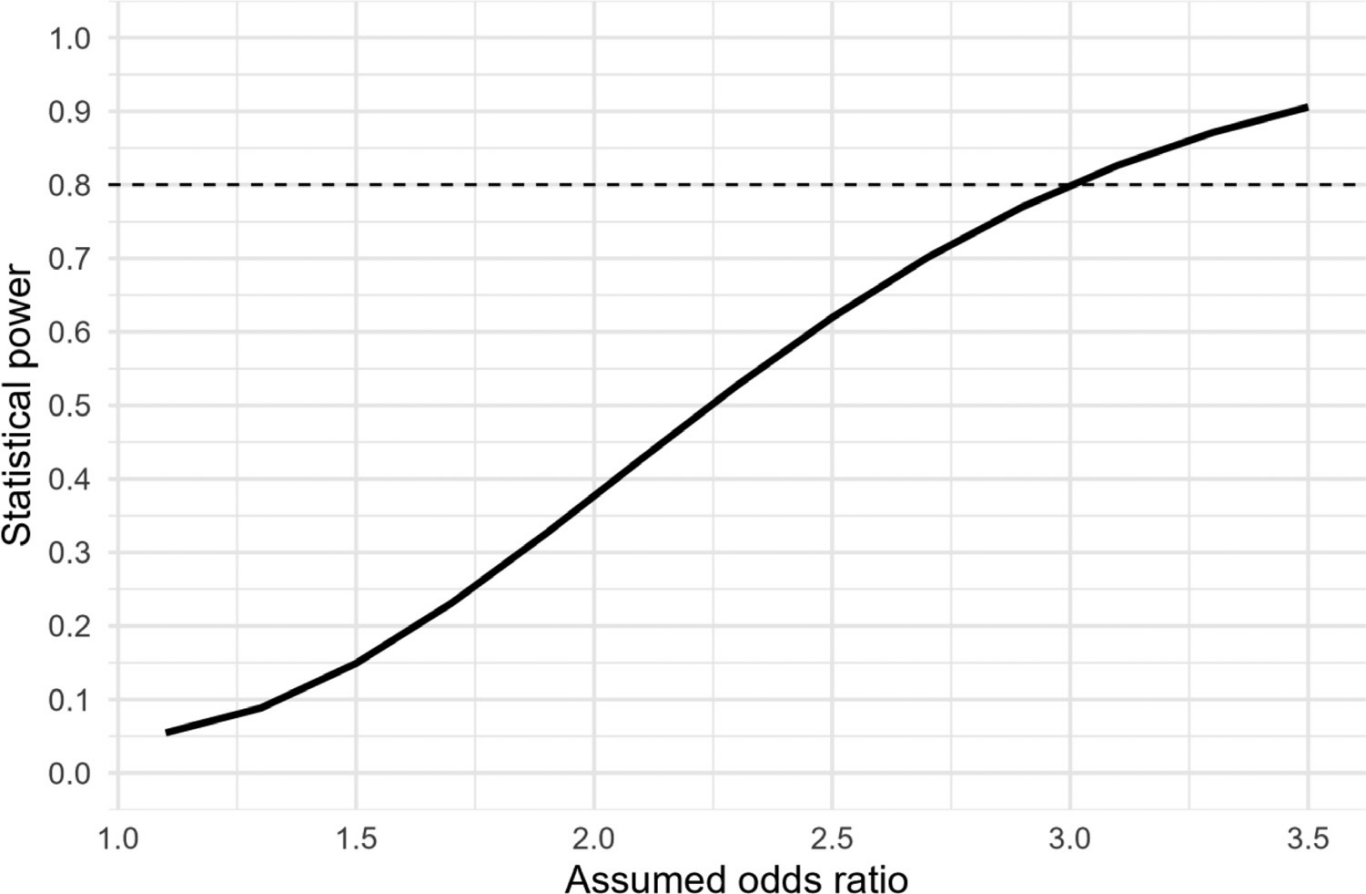
Power curve for detecting an association between rs17235409 and tuberculosis susceptibility under the observed minor allele frequency (MAF = 0.095) with 50 cases and 50 controls, illustrating statistical power across a range of assumed odds ratios at α = 0.05. MAF = minor allele frequency.

Figure 2 provides a graphical representation of the power analysis, showing the relationship between odds ratio and power for different sample sizes and MAF values.

## 4. Discussion

In this study, we investigated the influence of the SLC11A1 gene non-synonymous mutation rs17235409 (D543N) on tuberculosis susceptibility in a diverse ethnic population from southwestern China. Our results did not reveal a significant association between rs17235409 and tuberculosis susceptibility, either in the overall population or in specific ethnic subgroups. This finding is in contrast to some previous studies, which have reported a significant association between rs17235409 and increased TB risk in certain populations,^[19,20]^ but aligns with others that have found no significant association.^[22,23]^

The SLC11A1 gene has been extensively studied for its role in TB susceptibility due to its crucial involvement in macrophage activation and innate immunity against intracellular pathogens.^[8,9]^ The rs17235409 polymorphism, resulting in an amino acid change from aspartic acid to asparagine at position 543, has garnered particular attention because of its potential functional implications. This mutation occurs in a region of the protein involved in the transport of divalent cations, and the change from a negatively charged to a neutral amino acid could potentially affect the protein’s function or interaction with other molecules.^[24,25]^

The lack of association between rs17235409 and TB susceptibility observed in our study could be interpreted in several ways. First, it’s possible that this polymorphism does not significantly influence TB susceptibility in the specific ethnic groups studied. The functional impact of the D543N change might not be substantial enough to affect the protein’s ability to control *M tuberculosis* infection, or the effect might be compensated by other genetic or environmental factors.

Second, the minor allele frequency (MAF) of rs17235409 in our study population was 0.095, which is lower than the MAF of 0.149 reported for the Han Chinese in Beijing (CHB) population in the 1000 Genomes Project. This difference could be due to the diverse ethnic composition of our study population, which included Miao, Dong, and other ethnic groups from southwestern China. The lower MAF might have limited our ability to detect significant associations, particularly if the effect size is modest.

Third, our study’s sample size (50 cases and 50 controls) might not have provided sufficient statistical power to detect modest genetic effects. Power calculation revealed that our study had 80% power to detect an odds ratio of 2.5 or greater. If the true effect of rs17235409 on TB susceptibility is more modest, a larger sample size would be required to detect it.^[26-29]^

Given the low MAF observed in this cohort and the limited sample size, our study had adequate power only to detect relatively large genetic effects. Most susceptibility loci identified in TB genetic studies exhibit ORs ranging from 1.1 to 1.5. Therefore, the absence of statistically significant associations in this study should be interpreted cautiously, as modest effects may have remained undetected. Consequently, our findings should be regarded as preliminary evidence derived from a pilot investigation rather than definitive proof of no association.

Our finding of no association between rs17235409 and TB susceptibility aligns with some previous studies. For instance, Soborg et al found no significant association between this polymorphism and TB in a Danish population.^[30]^ Similarly, a meta-analysis by Li et al, while overall supporting an association between SLC11A1 polymorphisms and TB susceptibility, noted significant heterogeneity across studies and reported that the association might not be consistent across all populations.^[21]^

On the other hand, several studies have reported a significant association between rs17235409 and TB susceptibility. For example, a study by Liu et al in a Chinese population found that the A allele of rs17235409 was associated with an increased risk of TB.^[19]^ Similarly, Gao et al reported a significant association in a Han Chinese population.^[20]^ These contrasting findings highlight the complex nature of genetic susceptibility to TB and the importance of considering ethnic differences in genetic association studies. Unlike previous studies conducted primarily in Han Chinese populations, our study focused on ethnic minority groups from southwestern China, including Miao and Dong populations for which genetic evidence remains limited. Although no significant association between rs17235409 and tuberculosis susceptibility was observed, the present study provides preliminary information regarding allele and genotype distributions in these underrepresented populations. Such data may contribute to a broader understanding of ethnic heterogeneity in tuberculosis susceptibility and serve as a foundation for future multicenter investigations with larger sample sizes and more comprehensive genomic approaches.

Our stratification analysis by ethnicity did not reveal significant associations in any of the ethnic subgroups. In the Miao ethnic group, the odds ratio for the GA genotype compared to the GG genotype was 1.63 (95% CI: 0.23–11.46), suggesting a potential trend towards increased risk, but this was not statistically significant (*P* = 1.000). In the Dong ethnic group, the odds ratio was 0.62 (95% CI: 0.09–4.34), indicating a nonsignificant protective effect of the GA genotype. In the “Others” group, the genotype distributions were identical between cases and controls, resulting in an odds ratio of 1.00 (95% CI: 0.27–3.74). These findings suggest that the effect of rs17235409 on TB susceptibility might not vary significantly across the ethnic groups studied, although larger sample sizes would be needed to confirm this.

Given the relatively small sample size and the low minor allele frequency of rs17235409 in our study population, caution is warranted when interpreting this finding. The absence of AA homozygotes among cases may simply reflect limited statistical power rather than a true biological effect. Replication in larger, independent cohorts will be necessary to determine whether the A allele confers a modest protective influence against tuberculosis susceptibility in these ethnic populations.

Another observation from our study is the absence of the AA genotype for rs17235409 in both the case and control groups. This is not unexpected given the low MAF of 0.095 in our study population. With such a low allele frequency, the expected frequency of the AA genotype under Hardy–Weinberg equilibrium would be approximately 0.01, corresponding to about 1 individual in our total sample of 100. The absence of AA homozygotes is therefore not surprising and is consistent with the low MAF observed in our study.

Our study has several strengths. First, we included participants from diverse ethnic backgrounds, allowing us to investigate potential ethnic differences in the association between rs17235409 and TB susceptibility. Second, we employed rigorous quality control measures, including checks for Hardy–Weinberg equilibrium, high call rates, and duplicate sample testing, to ensure the reliability of our genotyping results. Third, we considered various genetic models to comprehensively evaluate the association between rs17235409 and TB susceptibility. Unlike previous studies conducted primarily in Han Chinese populations, our study focused on ethnic minority groups from southwestern China, including Miao and Dong populations for which genetic evidence remains limited. Although no significant association between rs17235409 and tuberculosis susceptibility was observed, the present study provides preliminary information regarding allele and genotype distributions in these underrepresented populations. Such data may contribute to a broader understanding of ethnic heterogeneity in tuberculosis susceptibility and serve as a foundation for future multicenter investigations with larger sample sizes and more comprehensive genomic approaches. Interestingly, the AA genotype was observed only among controls and was confined to the “Others” ethnic subgroup. Given the very small number of individuals carrying this genotype, this finding should be interpreted cautiously and requires confirmation in larger studies.

The absence of a statistically significant association between rs17235409 and tuberculosis susceptibility in the present study should be interpreted with caution. Several factors may explain these negative findings. First, insufficient statistical power is likely to be a major contributor. Based on the observed minor allele frequency (MAF = 0.135) and the available sample size, our study had adequate power only to detect relatively large genetic effects. However, most susceptibility loci identified in candidate gene studies and genome-wide association studies of tuberculosis confer modest effects, with odds ratios typically ranging from 1.1 to 1.5. Therefore, the current study may have been underpowered to detect small but biologically meaningful associations, increasing the possibility of a type II error. Second, residual confounding cannot be excluded. Although controls were selected from the same geographic region and frequency-matched according to ethnicity, significant differences in age and sex remained between the case and control groups. Because both demographic factors may influence immune responses and tuberculosis susceptibility, incomplete matching may have obscured true associations. In addition, the retrospective recruitment of participants from a single medical center may have introduced selection bias, thereby limiting the representativeness and generalizability of the findings. Third, the genetic architecture of tuberculosis susceptibility is highly heterogeneous across populations. Southwestern China is characterized by substantial ethnic diversity, and the allele frequencies of susceptibility loci may differ considerably among ethnic groups. The lower frequency of the A allele observed in our study compared with reference Han Chinese populations may have further reduced the ability to detect associations. Moreover, the relatively small number of participants within each ethnic subgroup limited the statistical power of stratified analyses. Finally, tuberculosis is a complex disease influenced by multiple host genetic variants, environmental exposures, and their interactions. The effect of a single polymorphism such as rs17235409 may therefore be insufficient to substantially alter disease risk in isolation. Potential gene–gene interactions involving other immune-related loci, as well as gene–environment interactions associated with nutritional status, socioeconomic conditions, smoking, and exposure intensity to Mycobacterium tuberculosis, were not evaluated in the present study and warrant further investigation. Taken together, these considerations suggest that the lack of statistically significant findings in this pilot study should not be interpreted as definitive evidence against a role for SLC11A1 in tuberculosis susceptibility. Rather, our results indicate that rs17235409 is unlikely to exert a large effect in this population, while more modest effects require validation in larger, multicenter studies with improved matching and comprehensive genetic analyses.

Future studies with larger sample sizes, more balanced case-control matching, and comprehensive genetic analysis of the SLC11A1 gene would be valuable to further elucidate the role of this gene in TB susceptibility. Additionally, functional studies investigating the molecular consequences of the D543N mutation would provide valuable insights into the mechanisms by which SLC11A1 variants might influence TB susceptibility.

## 5. Conclusion

In conclusion, our study found no significant association between the SLC11A1 gene non-synonymous mutation rs17235409 (D543N) and tuberculosis susceptibility in a diverse ethnic population from southwestern China. This finding was consistent across the overall population and specific ethnic subgroups. While our study had limited power to detect modest genetic effects, it contributes to the growing body of evidence on the role of SLC11A1 variants in TB susceptibility.

## Author contributions

**Conceptualization:** Chao Chen, Dianju Gu, Yunmi Xie.

**Data curation:** Chao Chen, Dianju Gu, Yunmi Xie.

**Formal analysis:** Chao Chen, Dianju Gu, Yunmi Xie.

**Funding acquisition:** Dianju Gu.

**Investigation:** Dianju Gu.

**Writing—original draft:** Dianju Gu.

**Writing—review & editing:** Dianju Gu.
